# Do seriously offending girls differ from their age- and offence type-matched male counterparts on psychopathic traits or psychopathy-related background variables?

**DOI:** 10.1186/s13034-016-0128-1

**Published:** 2016-11-01

**Authors:** Nina Lindberg, Svetlana Oshukova, Jouko Miettunen, Riittakerttu Kaltiala-Heino

**Affiliations:** 1 Forensic Psychiatry, Helsinki University and Helsinki University Hospital, Helsinki, Finland; 2 Psychiatry, Helsinki University and Helsinki University Hospital, Helsinki, Finland; 3 Research Unit for Clinical Neuroscience, Department of Psychiatry, University of Oulu and Oulu University Hospital, Oulu, Finland; 4 Medical Research Center Oulu, Oulu University Hospital and University of Oulu, Oulu, Finland; 5 Center for Life Course Health Research, University of Oulu, Oulu, Finland; 6 Adolescent Psychiatry, Tampere University and Tampere University Hospital, Tampere, Finland; 7 Kellokoski Hospital, Vanha Valtatie 198, 04500 Kellokoski, Finland; 8 Vanha Vaasa Hospital, Vaasa, Finland

**Keywords:** Adolescence, Delinquency, Gender, Girl, Offending, Psychopathic traits

## Abstract

**Background:**

More research is needed to improve our understanding of the manifestation of psychopathic traits in violently offending girls. Our aim here was to assess psychopathic traits and psychopathy-related background variables in a Finnish nationwide consecutive sample of girls charged with violent crimes and referred to a pretrial forensic psychiatric examination. These girls were then compared to their male counterparts.

**Methods:**

The forensic psychiatric examination statements of 15- to 17-year-old juveniles who underwent a pretrial forensic psychiatric examination over a 31-year period (1980–2010) were reviewed. For each non-psychotic girl with a normal IQ (n = 25), an age- and offence type-matched male control was randomly selected. Offence and offender characteristics were collected from the forensic psychiatric examination reports, and a file-based assessment of psychopathic traits was performed using the Hare psychopathy checklist-youth version.

**Results:**

Approximately every third girl exhibited high traits of psychopathy, and no significant difference was observed between the genders. Focusing on the underlying factor and item scores, the girls scored significantly lower than boys on the Antisocial factor. Their interpersonal relationships were significantly more unstable and they significantly more often exhibited a history of child sexual abuse. During the index offence the girls were significantly less frequently intoxicated, and their victims were significantly more often family members or current or ex-intimates but significantly less often strangers.

**Conclusions:**

Although violently offending girls and boys do not differ on psychopathy total scores, significant gender differences exist on underlying factor and item scores as well as in background- and offence-related variables. Interventions should take into account these special features of violent girls.

## Background

A personality trait is a fairly stable way of experiencing and perceiving oneself as well as perceiving and relating to others. Psychopathy is a constellation of interpersonal (dishonest charm, grandiosity, lying, and manipulative behavior) affective (remorselessness, unemotionality, and callousness) and behavioral (thrill-seeking, impulsivity, and irresponsibility) traits [1]. Current conceptualizations see psychopathic traits on a dimensional continuum, where psychopathy is a malicious version of the extremes of normal personality traits [2]. In 2013, a subtype of conduct disorder characterized by callous-unemotional traits was introduced in the fifth version of the diagnostic and statistical manual of mental disorders (DSM 5) [3]. The specifier “with limited prosocial emotions (LPE)” is used when an individual, suffering from conduct disorder, exhibits two or more of the following characteristics in multiple relationships or settings over a 12-month period: (1) lack of remorse or guilt, (2) callousness/lack of empathy, (3) shallow or deficient affect, and (4) unconcern about his/her performance. Psychopathic traits are relatively stable over time, from childhood through adolescence to adulthood [4, 5]. On the other hand, traits can change across favorable development and treatment [6]. Research findings on gender differences in psychopathic traits in adolescence are mixed, with some studies reporting overall higher psychopathic tendencies among boys than among girls, and others finding no gender difference [7]. Higher psychopathy scores for boys than for girls tend to emerge in samples recruited from community settings, while studies among institutionalized youth have reported fewer differences in psychopathic scores across the genders [7]. Evidence also indicates different expression of psychopathy in girls and boys where, among girls, the interpersonal and affective features more clearly capture psychopathy than do the behavioral ones [8].

In light of current literature, psychopathy is strongly associated with genetic and neurobiological background [9], but also environmental factors seem to have an influence on its development [10]. Broken families, institutional or foster home placements, low parental care, harsh discipline, physical and psychological abuse, parents’ antisocial and criminal behavior, parents’ mental health and substance use problems, delinquent siblings, large family size, school difficulties, and mental health problems in childhood and/or adolescence are all associated with elevated psychopathic traits [11–16]. The literature suggests that there are differences in the origins of and developmental pathways to psychopathy between the genders [17]. Evidence supports a lower heritability of psychopathic traits [18–20] as well as a greater role of family-related risk factors in girls than in boys [21]. However, according to a recent study by Ficks et al. [22], the etiology of psychopathic traits in youth seems to be highly similar for girls and boys.

Juvenile psychopathy is becoming an increasingly important construct in judicial systems [17]. Offenders with strong psychopathic traits typically begin their antisocial and criminal activities at a relatively young age and continue to engage in these activities throughout their lifespan [23]. In addition, their use of violence tends to be more instrumental, dispassionate, and predatory than that of other offenders [24]. They also re-offend more quickly and more often following release from custody than do other offenders [25, 26]. Elevated traits of psychopathy relate to severity of violence [27], and affective-interpersonal features of psychopathy predict more frequent use of sadistic violence, repeated violence against the same victim, and violence resulting in more serious victim injuries [28]. Psychopathic traits associate with a greater likelihood of an adolescent to offend in groups and to be in a gang, as well as to take a leadership role in group crimes [29].

The number of studies focusing on psychopathic traits in offending girls is limited, most probably due to the small number of female offenders overall [30, 31], yet they seem to represent a growing population [32]. In a study by Schrum and Salekin [8] among 123 girls aged 11–18 years from two American detention centers, as many as 16.9 % exhibited significant traits of psychopathy (the psychopathy checklist—youth version [PCL-YV] > 25). In a study by Campbell et al. [33] among 226 Canadian incarcerated 12- to 19-year-old youngsters (girls: 17 %), girls and boys did not significantly differ from each other in psychopathic traits. In this sample, the most frequent convictions in both genders were non-violent offences (86 %) and the base rate of high psychopathic traits (PCL-YV > 25) was relatively low (9.4 %). Accordingly, no gender difference was reported by Salekin et al. [34] among 114 children and adolescents (girls: 29.8 %) recruited from an American detention center. The crimes of these youngsters included mainly thefts, assaults, and other violent offences. However, in a German sample of 90 incarcerated boys and 123 girls aged 14- to 19 years, boys exhibited significantly higher traits of psychopathy (both total and underlying factor scores) than did girls [35]. In this sample, 71.6 % of the adolescents had been convicted of at least one violent criminal act. With regard gender differences, 36.3 % of the detained girls had never been convicted of a violent crime versus only 20.2 % of the boys. Further, in a Canadian sample of 142 youngsters (76 boys and 66 girls) aged 12- to 18 years, who had been actively involved in the local criminal justice system and/or who had been diagnosed as having a severe conduct disorder, boys exhibited significantly higher affective-interpersonal traits of psychopathy than did girls, but no gender difference was observed on the behavioral component of psychopathy [36]. In this sample, the vast majority (96 %) reported being involved in at least one violent act in the course of their lives. Girls reported engaging in significantly fewer types of violent crimes than did boys. With regard to non-violent offences, no statistically significant gender difference was observed.

To summarize, there is a need for further research to improve our understanding of the manifestation of psychopathic character traits in offending girls. The aim of this study was to assess psychopathic traits as well as psychopathy-related background variables in a Finnish nationwide sample of juvenile girls charged with violent offences and referred to a pretrial forensic psychiatric examination and to compare these girls with their age- and offence-matched male counterparts. Our hypotheses were that we would find a subgroup of girls exhibiting high traits of psychopathy and that the gender differences would be minimal in this special population of highly violent underaged criminals.

## Sample and methods

### Sample and procedure

Information was obtained from the National Institute of Health and Welfare, which organizes the forensic psychiatric examinations in Finland. According to Finnish law, courts decide whether a forensic examination is needed. After deciding on the examination, the court asks the National Institute of Health and Welfare to arrange it. Forensic psychiatric examinations are inpatient evaluations lasting approximately 2 months, and include data gathered from various sources (family members, relatives, and medical, criminal, school, child welfare, and military records), psychiatric evaluation, standardized psychological tests, interviews conducted by a multi-professional team, evaluation of the offender’s physical condition, and continuous observation of the offender by hospital staff. The final forensic psychiatric report includes an opinion on the level of criminal responsibility, a possible psychiatric diagnosis, and an assessment as to whether or not the offender fulfils the criteria for involuntary psychiatric care. The overall quality and reliability of Finnish forensic psychiatric examinations are considered high by both courts and scientists [37]. In Finland, psychiatric classification according to the International Classification of Diseases—Eighth Revision (ICD-8) [38] served in clinical practice between 1968 and 1986 and was replaced by the diagnostic and statistical manual of mental disorders, third edition, revised (DSM-III-R) [39], which was used between the years 1987 and 1995. Since 1996, ICD-10 [40] has been in use.

In Finland, the minimum age of criminal liability is 15 years. As part of a large research project on Finnish juvenile pretrial offenders, forensic psychiatric examination reports of all 15- to 17-year-old offenders who underwent the examination in 1.1.1980–31.12.2010 were collected from the archives of the National Institute of Health and Welfare. Forensic psychiatric examination reports were retrospectively reviewed. The study protocol was approved by the Ethics Committee of Helsinki University Hospital and the pertinent institutional authorities.

### Assessment of psychopathic traits

The psychopathy checklist—youth version (PCL-YV) [41], which is an adaptation of the psychopathy checklist—revised (PCL-R) [42], was used to assess psychopathic traits. Each of the 20 PCL-YV items is rated as 0 (absent), 1 (present to some degree or contradictory data), 2 (definitely present), or omitted if the information was insufficient. To aid in the scoring and determination of each trait, the manual provides an item description and some behavioral examples [41]. An individual’s assessment is rejected if it contains more than five omitted items [41]. The total score can range from 0 to 40, with higher scores reflecting a greater number of psychopathic traits. The total PCL score is dimensional, but in research settings categorical diagnoses are used as well. There is no recommended cut-off score for use with the PCL-YV, but on the PCL-R total scores ranging from 30 to 40 are considered diagnostic of psychopathy [43]. In line with recommendations of a lower cut-off score for European populations [44–46], a cut-off score of 25 has been used in studies performed in Scandinavian countries [47–49] and a score of 20 is sometimes considered to be a cut-off for “medium psychopathy” [50]. The PCL-YV items can be summed to yield four factors: factor I or the interpersonal factor (items: impression management, grandiose sense of self-worth, pathological lying, manipulation for personal gain), factor II or the affective factor (items: lack of remorse/guilt, shallow affect, callous/lack of empathy, failure to accept responsibility), factor III or the behavioral factor (items: stimulation-seeking, parasitic orientation, lack of goals, impulsivity, irresponsibility), and factor IV or the antisocial factor (items: poor anger control, early behavior problems, serious criminal behavior, serious violation of conditional release, criminal versatility). Although PCL assessments should be based on both a review of file information and a semi-structured interview, several studies have shown that PCL assessments can reliably be made for both adults [51–54] and adolescents [28, 55–58] without the interview when sufficient file information is available. In this study, the scoring was done by one officially trained female rater (NL). After this, 20 (40 %) reports were randomly chosen and rated by one independent male rater to assess inter-rater reliability. The inter-rater agreement was assessed using intraclass correlation (ICC). The ICC was 0.886 for PCL-YV total score, 0.748 for factor 1 score, 0.890 for factor 2 score, 0.868 for factor 3 score and 0.844 for factor 4 score. All correlations were significant (p < 0.001). The internal consistency, as measured by Cronbach’s alpha, was 0.89 for all items, 0.86 for factor 1, and 0.79 for factor 2, 0.84 for factor 3, and 0.74 for factor 4.

### Offender- and offence-related variables

Data on demographics, family-related characteristics [parents’ divorce, parental psychiatric and substance use problems, family size (more than four children in the family), institutional/foster home placements, criminality of near relatives], problems related to school (failure to pass grade in primary/secondary schools, attending special education), psychiatric (mental health contacts), social (client of social services, witnessing/subject of physical violence at childhood home, sexual abuse), and criminal history (previous non-violent and violent offending and the index offence (intoxication, more than one victim, more than one offender, victim-offender relationship [family or (ex-)intimate, acquaintance, stranger], signs of instrumental violence were gathered from the forensic psychiatric evaluation reports. All items were rated categorically as 1 (=present) or 0 (=absent). After one rater (NL) had coded all the cases, 20 (40 %) randomly selected reports were coded by an independent male rater in order to ensure that variables were unambiguous enough to guarantee reliable subjective interpretations of their presence/absence. Interrater agreement was assessed using Cohen’s kappa (κ) [59]. According to the Landis and Koch guidelines [60], all variables showed substantial (0.61–0.80) or almost perfect (0.81–1) agreement.

### Statistical analysis

We conducted a multivariate analysis (MANOVA) across the four factors of the PCL-YV. We continued with the Independent samples t test, the Likelihood ratio Chi square test, and Fisher’s exact test to compare the groups. Findings were considered significant when p < 0.05. The phi (φ) coefficient was used as an effect size measure for the Chi square test, and Cohen’s d for the Independent samples t test. The magnitude of the φ coefficient was interpreted as follows: 0.1 as small, 0.3 as moderate, and 0.5 as large effect, and respectively, Cohen’s d as follows: 0.2 as small, 0.5 as moderate, and 0.8 as large effect [61]. We conducted data analyses with the SPSS statistical software package version 22.

## Results

During the study period altogether 266 adolescents aged 15–17 years underwent a forensic psychiatric examination, 29 (10.9 %) of whom were girls. Of these juveniles, 4 girls and 21 boys suffered from intellectual disability or a current psychotic disorder (mainly schizophrenia) and were excluded from further analyses since it is questionable whether a youngster with abnormally low IQ or acute symptoms of psychosis can be scored with the PCL-YV. The remaining 25 girls with a mean age of 16.3 (SD 0.74) years were all native Finns and charged with violent offences, including murder (n = 5), attempted murder (n = 4), manslaughter (n = 2), attempted manslaughter (n = 7), aggravated assault (n = 2), arson (n = 3), and violent robbery (n = 2). Nineteen (76.0 %) of them showed a history of previous offending and 8 (32.0 %) had a history of violent offending (mainly assaults) before the index crime. Twenty (80 %) girls had been arrested before the index offence, but none of them had been imprisoned. Most girls were diagnosed with a conduct disorder or a personality disorder (n = 21), but four girls had no mental disorder. For each girl, an age- and offence type-matched male control of Finnish origin was randomly selected from the national data.

The comparisons between girls and boys are presented in Table 1. Approximately every third girl exhibited significant (PCL-YV > 25) psychopathic traits, and no significant difference was observed between the genders. MANOVA revealed significant gender differences in factor structure (Wilk’s Λ = 0.733, F (4.45) = 4.10, p = 0.006, η^2^ = 0.27). Girls scored significantly lower on the antisocial factor, including poor anger control, early behavioral problems, and criminal versatility, than boys. Girls also scored lower on stimulation-seeking, impulsivity, and irresponsibility, which load onto the Behavioral factor. There was a tendency for girls to score higher on the affective factor, but the difference did not reach statistical significance. Girls scored significantly higher on the item “unstable interpersonal relationships”, and they also showed a tendency to score higher on the item “Impersonal sexual behavior”.

**Table 1:** Comparisons between a nationwide sample of girls charged with violent offences and referred to a pretrial forensic psychiatric examination (n = 25) and their age- and offence-matched male counterparts

	Girls	Boys	Statistics^a^	p	Effect size
PCL-YV					
Total score mean (SD)	19.76 (8.52)	20.32 (6.50)	t = −0.261	0.795	d = −0.073
Factor 1 (interpersonal)	1.56 (2.18)	1.68 (1.86)	t = −0.209	0.835	d = −0.060
Factor 2 (affective)	4.44 (2.38)	3.24 (2.33)	t = 1.793	0.079	d = 0.518
Factor 3 (behavioral)	6.44 (2.83)	7.64 (1.87)	t = −1.770	0.083	d = 0.511
Factor 4 (antisocial)	5.52 (2.62)	6.88 (1.74)	t = −2.164	*0.035*	d = 0.624
Total score > 30	3/25 (12.0 %)	3/25 (12.0 %)	#	1.000	NA
Total score > 25	8/25 (32.0 %)	7/25 (28.0 %)	X^2^ = 0.095	0.758	φ = −0.044
Total score > 20	16/25 (64.0 %)	14/25 (56.0 %)	X^2^ = 0.333	0.564	φ = −0.082
Item mean (SD)					
Impression management	0.44 (0.58)	0.48 (0.51)	t = −0.209	0.795	d = 0.060
Grandiose sense of self-worth	0.32 (0.56)	0.40 (0.65)	t = 0.469	0.641	d = 0.135
Stimulation-seeking	1.44 (0.71)	1.92 (0.28)	t = −3.142	*0.003*	d = 0.907
Pathological lying	0.32 (0.69)	0.40 (0.76)	t = −0.389	0.699	d = 0.113
Manipulation for personal gain	0.52 (0.77)	0.36 (0.70)	t = 0.769	0.446	d = 0.222
Lack of remorse/guilt	1.32 (0.75)	0.92 (0.76)	t = 1.876	0.067	d = 0.542
Shallow affect	0.88 (0.83)	0.72 (0.69)	t = 0.745	0.460	d = 0.215
Callous/lack of empathy	1.00 (0.71)	0.72 (0.61)	t = 1.495	0.141	d = 0.432
Parasitic orientation	0.64 (0.76)	0.84 (0.80)	t = −0.908	0.368	d = 0.262
Poor anger control	1.36 (0.70)	1.84 (0.37)	t = −3.024	*0.004*	d = 0.873
Impersonal sexual behavior	0.80 (0.96)	0.36 (0.64)	t = 1.912	0.062	d = 0.552
Early problem behavior	1.20 (0.91)	1.68 (0.56)	t = −2.245	*0.039*	d = 0.648
Lack of goals	1.52 (0.71)	1.40 (0.76)	t = 0.574	0.569	d = 0.166
Impulsivity	1.56 (0.71)	1.92 (0.28)	t = −2.357	*0.023*	d = 0.680
Irresponsibility	1.28 (0.69)	1.64 (0.57)	t = −2.034	*0.048*	d = 0.587
Failure to accept responsibility	1.24 (0.60)	0.88 (0.73)	t = 1.915	0.061	d = 0.553
Unstable interpersonal relationships	1.00 (0.96)	0.28 (0.61)	t = 3.166	*0.003*	d = 0.914
Serious criminal behavior	1.48 (0.51)	1.60 (0.50)	t = −0.840	0.405	d = 0.242
Serious violation of conditional release	0.48 (0.65)	0.32 (0.48)	t = 0.990	0.327	d = 0.286
Criminal versatility	1.00 (0.82)	1.48 (0.71)	t = −2.213	*0.032*	d = 0.639
Background variables					
Childhood (0–12 years) home					
Parents’ divorce	16/25 (64.0 %)	17/24 (70.8 %)	X^2^ = 0.260	0.610	φ = 0.073
Mother’s substance use problems	10/25 (40.0 %)	5/25 (20.0 %)	#	0.217	NA
Father’s substance use problems	14/24 (58.3 %)	17/24 (70.8 %)	X^2^ = 0.820	0.365	φ = 0.131
Mother’s psychiatric problems	5/25 (20.0 %)	8/25 (32.0 %)	#	0.520	NA
Father’s psychiatric problems	3/24 (12.5 %)	6/24 (25.0 %)	#	0.461	NA
Witnessing physical violence	13/25 (52.0 %)	12/25 (48.0 %)	X^2^ = 0.080	0.777	φ = −0.400
Subject of physical violence	15/25 (60.0 %)	9/25 (36.0 %)	X^2^ = 2.885	0.089	φ = −0.240
More than four children in the family	11/25 (44.0 %)	9/25 (36.0 %)	X^2^ = 0.333	0.564	φ = −0.082
Institutional/foster home placement	7/25 (28.0 %)	10/25 (40.0 %)	X^2^ = 0.802	0.370	φ = 0.127
Other childhood (0–12 years) variables					
Subject of sexual abuse	9/25 (36.0 %)	0/25 (0.0 %)	#	*0.001*	NA
Mental health contact	8/25 (32.0 %)	9/25 (36.0 %)	X^2^ = 0.089	0.765	φ = 0.042
Client of social services	15/25 (60.0 %)	18/25 (72.0 %)	X^2^ = 0.802	0.370	φ = 0.127
Criminality of near relatives					
Mother’s criminality	1/25 (4.0 %)	0/25 (0.0 %)	#	1.000	NA
Father’s criminality	7/24 (28.0 %)	8/24 (32.0 %)	X^2^ = 0.097	0.755	φ = 0.045
Delinquent sibling/s	8/25 (32.0 %)	10/25 (40.0 %)	X^2^ = 0.347	0.556	φ = 0.083
Homicide history of parents or near relatives	5/25 (20.0 %)	2/25 (8.0 %)	#	0.417	NA
School performance in primary and secondary schools					
Failure to pass grade at school	14/25 (56.0 %)	14/25 (56.0 %)	X^2^ = 0.082	0.774	φ = −0.041
Attending special education	11/25 (44.0 %)	14/25 (56.0 %)	X^2^ = 0.720	0.396	φ = 0.120
Offence-related variables					
History of previous offending	19/25 (76.0 %)	21/25 (84.0 %)	X^2^ = 0.500	0.480	φ = 0.100
History of violent offending	8/25 (32.0 %)	12/25 (48.0 %)	X^2^ = 1.333	0.246	φ = 0.163
Current alcohol use disorder	15/25 (60.0 %)	15/25 (60.0 %)	X^2^ = 0.000	1.000	φ = 0.000
Intoxicated during index offence	15/25 (60.0 %)	22/25 (88.0 %)	X^2^ = 5.094	*0.024*	φ = 0.319
More than one offender	15/25 (60.0 %)	10/25 (40.0 %)	X^2^ = 2.000	0.157	φ = −0.200
More than one victim	3/25 (12.0 %)	5/24 (20.8 %)	#	0.463	NA
Victim-offender relationship					
Family or (ex-) intimate	9/24 (37.5 %)	0/24 (0.0 %)	#	*0.001*	NA
Acquaintance	11/24 (45.8 %)	5/24 (20.8 %)	#	0.062	NA
Stranger	4/24 (16.7 %)	19/24 (79.2 %)	#	<0.001	NA
Index offence characterized with instrumental violence	7/25 (28 %)	9/25 (36 %)	X^2^ = 0.368	0.544	φ = 0.086

Independent samples t test (t), the Chi square test (χ^2^), and the Fisher’s exact test (#) are used for comparing the groups. Effect sizes are reported: *d* Cohen’s d, φ = phi

Statistically significant differences are in italics

*NA* not applicable

With regard to background variables, girls had been significantly more often sexually abused as children than boys. There was also a tendency for girls to more often have been subjects of physical violence in the childhood home. With regard to offender- and offence-related variables, girls were significantly less often intoxicated during the index offence, and their victims were significantly more often family members or current or ex-intimates but less often strangers.

## Discussion

An important question is if, and if so, to what extent psychopathic traits manifest differentially as a function of gender. In this nationally representative sample of highly violent adolescents, girls and boys who had committed comparable crimes were also comparable regarding their psychopathic traits; about two-thirds of both genders exhibited elevated psychopathic traits and one-third showed high traits of psychopathy. This is in line with the findings reported by Campbell et al. [33] and Salekin et al. [34] who found equal rates of psychopathy among incarcerated girls and boys. Our findings contradict the findings of Sevecke et al. [35] and Penney et al. [36] who reported that boys in criminal justice processes exhibited higher psychopathic trait scores than girls. In both these studies, however, boys had a history of more numerous violent offences than girls. The gender difference in severity of offending likely contributes to the differences seen on psychopathy scores, as in our sample the genders were matched for their crimes. Further reasons explaining the different finding could be differences in psychopathology other than psychopathy, and perhaps differences in histories of subjection to abuse and trauma. So, it is obvious that the roles of psychopathology and trauma deserve further research. Our findings suggest that those girls who commit equally severe violent crimes than boys also display psychopathic traits to equal extend. Girls with less severe (violent) crimes likely display less psychopathic traits.

Even if girls and boys scored equally high on the PCL-YV total scores, girls scored significantly lower than boys on the antisocial factor. There was also a tendency for girls to score higher on the affective factor and for boys to score higher on the behavioral one, although these differences did not reach statistical significance. These findings differ from those reported by Penney et al. [36], where boys scored higher on both Interpersonal and Affective factors. Penney et al. used a three-factor model of the PCL-YV, excluding largely those items that in the model used here load onto the antisocial factor. Again, the differences between Penney et al.’s report and ours are likely due to the fact that in their sample boys were more violent than girls.

On an item-level, boys expressed significantly higher levels of stimulation-seeking, impulsivity, and irresponsibility, as well as poor anger control, early emotional problems, and criminal versatility, reflecting an antisocial lifestyle, and, diagnostically speaking, an antisocial personality disorder, which is often diagnosed in adult male prisoners [3]. Compared with boys, girls’ relationships showed significantly higher instability, which is one of the core symptoms of borderline personality, a disorder overrepresented among incarcerated women [62]. Although psychopathy is often represented as a unitary construct, it shows heterogeneity with different subtypes and multiple underlying trait dimensions. In adults, preliminary findings indicate an antisocial personality disorder variant as well as a borderline personality disorder variant of psychopathy exist, the latter being more prevalent in females [63]. Our results suggest that same kinds of gender-specific trait variations exist in adolescents. To explore this with cluster analysis methods, considerably larger sample sizes are needed. The authors are aware of one recent study focusing on latent class analyses based on self-assessed general personality traits among 12- to 17-year-old delinquents [64]. The study, based on the quick big five, suggested three personality types, consisting of an emotionally labile, close-minded and goal-oriented class, an under controlled class, and an emotionally labile-careless class. Interestingly, youngsters characterized with both emotional lability and carelessness scored highest on the externalizing behavior including rule-breaking and aggressive behavior as well as on the impulsive-irresponsible factor of the Youth Psychopathic traits Inventory. The authors, however, found no statistically significant gender differences within different personality classes. It is somewhat difficult to compare our results to those of Decuyper et al. [64], since Finnish forensic psychiatric statements typically include thorough descriptions of personality traits related to non-adjustment, but the descriptions of other personality traits remain often limited.

The family backgrounds of our pretrial adolescents were highly troublesome. Findings from studies investigating adverse childhood experiences indicate that they are highly correlated with each other [65], and various kinds of maltreatment are usually experienced simultaneously [66], as was the case here. Compared with a Finnish patient sample collected from one of the two national forensic units treating adolescents with severe mental disorders and violent behaviors [67], the families of pretrial offenders were even more commonly characterized by parental divorce, substance use, and criminality, suggesting an intergenerational cycle of criminality. Despite having being in contact with child welfare services, the offender sample had less commonly been institutionalized or placed in foster homes than the patient sample, so they most probably had been exposed for longer to harmful adverse childhood experiences.

Focusing on gender differences in background variables, only victimization by sexual abuse reached statistical significance. In fact, more than one in three girls, but none of the boys had reported sexual abuse in childhood, which is in line with earlier research reporting that subjection to sexual abuse is more common among female than male young offenders [68]. Victimization by sexual abuse in childhood is associated with a variety of emotional and behavioral problems, including violence, in both population and institutionalized samples [68–71]. It predisposes to repeated sexual trauma, drifting repeatedly into emotionally unsatisfactory and abusive relationships, and being incapable of protecting oneself from sexual health problems [72]. The violently offending girls in this sample indeed displayed unstable interpersonal relationships as well as impersonal sexual behavior more commonly than boys. Odgers et al. [73] have suggested that among high-risk girls victimization by abuse might be a better predictor for future violence than psychopathic traits, and that victimization may actually be an etiological factor for elevated psychopathic traits, or traits that mimic psychopathy (particularly deficient affect). Our findings support this speculation, and suggest that victimization in the sexual domain could be particularly distorting for female adolescent development.

Alcohol use disorders were common in our sample, and the majority of adolescents committed the index crime under the influence of alcohol, which is known to characterize Finnish homicide crimes performed by both adults [74] and adolescents [16]. Boys were, however, significantly more often than girls under alcohol intoxication during the index crime. This same gender difference has been reported in adults [75]. In line with the reports from the Institute of Criminology and Legal Policy in Finland [76, 77], victims of girls were often family members or current or previous intimates, and, respectively, those of boys were strangers. It is known that women typically carry out their crime in the context of home, and the violence is bound to their close relationships [75, 78]. To conclude, gender differences observed in our sample resemble in many ways those reported in adult offenders.

### Strengths and limitations

As far as the authors know, this is the first study to explore gender differences on psychopathic traits in girls and boys matched for both age and crime. The main strength of this study was its nationwide and comprehensive nature. The Finnish tradition of thorough forensic psychiatric examinations and reliable statistics form a solid basis for register-based studies. In Finland, the clearance rate for violent crimes is high; for example, the mean clearance rate for homicides during 1995–2004 was 92 % [79]. The proportion of females in our sample corresponds quite well with proportion of females in violent crimes, as reported in national statistics of police investigated crime and self-reported juvenile delinquency [77]. The assessments were performed with the PCL-YV, which is regarded as a gold standard for assessing adolescent psychopathic traits. It is a time-consuming method demanding rigorous training, but is more objective than self-report instruments, which are more or less transparent, and thus, inadequate in studying offender populations [80, 81]. Unfortunately, it was not possible to estimate the representativeness of our sample by comparing the number of girls who underwent the pretrial examination with the overall number of girls with violent offending during the 31-year period. However, a previous Finnish study estimated that approximately 60 % of 15- to 17-year-old homicide suspects undergo forensic psychiatric examination [82]. Regardless of the fact that the sample contained all Finnish under aged girls who underwent a forensic psychiatric examination during the 31-year period, the sample size remained small. Despite the high quality and reliability of Finnish forensic psychiatric examinations, the data is not collected primarily for research purposes. The information is not written down in any systematic manner. This means that it was not possible to infer reliably whether the missing data indicated that the variable was actually absent or that the presence of the variable was not written down in the statement. One possible methodological problem is the use of self-reports and reports from relatives in the forensic psychiatric examination statements. Examinees and parents might be reluctant to correctly report adverse conditions or odd behaviors from offender’s childhood. This might lead to under detection of, for example, incidences of mental illness or criminality in the family [83]. On the other hand, as Cannon et al. [84] pointed out, there might be a problem with recall bias when using retrospective data such as mother’s knowledge of child’s early development: the knowledge of child’s adult outcome may influence memories of childhood behavior. Despite these obvious limitations, however, forensic psychiatric examinations are a unique source of information in research on psychopathology and criminality. Taking into account the small sample size, we did not use corrections for multiple testing, but, instead, effects sizes were calculated. With small sample sizes, a difference may be clinically meaningful despite failing to achieve statistical significance. In this study, the fairy large effect sizes hint to this possibility.

## Conclusion

Although violently offending girls and boys did not differ in psychopathy total scores, significant gender differences existed on underlying factor and item scores as well as in background- and offence-related variables. Girls were less antisocial, but their relationships were more unstable, and their violence was bound to their close relationships. They also more often reported a history of child sexual abuse. Interventions with violent girls should focus on sexual trauma, appropriate and constructive skills to protect oneself against sexual abuse, and social skills in intimate relationships.
